# An ensemble-based feature selection framework to select risk factors of childhood obesity for policy decision making

**DOI:** 10.1186/s12911-021-01580-0

**Published:** 2021-07-21

**Authors:** Xi Shi, Gorana Nikolic, Gorka Epelde, Mónica Arrúe, Joseba Bidaurrazaga Van-Dierdonck, Roberto Bilbao, Bart De Moor

**Affiliations:** 1 Department of Electrical Engineering (ESAT), Stadius Centre for Dynamical Systems, Signal Processing and Data Analytics, KU Leuven, Kasteelpark Arenberg 10 - box 2446, 3001 Leuven, Belgium; 2 Vicomtech Foundation, Basque Research and Technology Alliance (BRTA), Donostia-San Sebastián, Spain; 3 Biodonostia Health Research Institute, eHealth Group, Donostia-San Sebastián, Spain; 4 Regional office of the Health Department, Basque Government, Bilbao, Spain; 5 Basque Foundation for Research and Innovation, Bilbao, Spain

**Keywords:** Feature selection, Ensemble learning, Childhood obesity, Public health, Policy decision making

## Abstract

**Background:**

The increasing prevalence of childhood obesity makes it essential to study the risk factors with a sample representative of the population covering more health topics for better preventive policies and interventions. It is aimed to develop an ensemble feature selection framework for large-scale data to identify risk factors of childhood obesity with good interpretability and clinical relevance.

**Methods:**

We analyzed the data collected from 426,813 children under 18 during 2000–2019. A BMI above the 90th percentile for the children of the same age and gender was defined as overweight. An ensemble feature selection framework, Bagging-based Feature Selection framework integrating MapReduce (BFSMR), was proposed to identify risk factors. The framework comprises 5 models (filter with mutual information/SVM-RFE/Lasso/Ridge/Random Forest) from filter, wrapper, and embedded feature selection methods. Each feature selection model identified 10 variables based on variable importance. Considering accuracy, F-score, and model characteristics, the models were classified into 3 levels with different weights: Lasso/Ridge, Filter/SVM-RFE, and Random Forest. The voting strategy was applied to aggregate the selected features, with both feature weights and model weights taken into consideration. We compared our voting strategy with another two for selecting top-ranked features in terms of 6 dimensions of interpretability.

**Results:**

Our method performed the best to select the features with good interpretability and clinical relevance. The top 10 features selected by BFSMR are age, sex, birth year, breastfeeding type, smoking habit and diet-related knowledge of both children and mothers, exercise, and Mother’s systolic blood pressure.

**Conclusion:**

Our framework provides a solution for identifying a diverse and interpretable feature set without model bias from large-scale data, which can help identify risk factors of childhood obesity and potentially some other diseases for future interventions or policies.

**Supplementary Information:**

The online version contains supplementary material available at 10.1186/s12911-021-01580-0.

## Background

Childhood obesity has emerged as an important public health problem all around the world. According to the WHO [1], the worldwide prevalence of obesity nearly doubled during 1980–2008 and one in three 11-year-old children is overweight or obese in Europe. A childhood obesity review has shown that the increasing prevalence of childhood obesity is associated with the emergence of comorbidities previously considered as “adult” diseases such as diabetes and hypertension which can track into adulthood [2]. Basque Government proposes the creation of specific childhood obesity prevention plan as one of the new two main action areas and targets to create regional policy for the Basque Region [3]. Therefore, it is essential to study the risk factors of childhood obesity to design preventive policies or interventions.

The increasing prevalence of childhood obesity is the consequence of an interaction among a complex set of factors that are related to the genetics (e.g. monogenic disorders, endocrine issues, etc.) and environmental effects (e.g. parental feeding styles, microbiota, school, society trend, etc.) [2]. There are some surveys capturing the reality of overweight and obese children, however, most of the surveys and studies only included hundreds or thousands of participants. Based on the summary statistics from the systematic reviews [4–6], very limited studies had more than 100,000 or even over one million participants. The rapid development of electronic health records (EHR) systems and abundant data from various sources make it possible to have access to real-world data on a larger scale. A review in 2018 explored the obesity studies using big data collected from different sources [7], such as social media, smartphones and healthcare wearable devices, transportation and so on. But these data samples had their own limitations, for example sample bias, ethical issues, or lack of linkage with nutrition information. A recent study used EHR data to predict the risk of childhood obesity, including almost one million participants [8], however, because of the characteristics of data sources, the features were all clinical variables, the environmental factors related with family and school were not included. Therefore, the Osakidetza database in Basque region can be of great value as it is large scale data covering millions of participants and includes specific information on different aspects of environmental factors of childhood obesity at the same time.

Studies on the risk factors of childhood obesity and interventions have been conducted and reported in the literature [2, 4, 5]. But difficulties still exist when designing preventive policies or interventions for a specific region. Despite that the risk factors given by different studies were similar, the variables used for each risk factor could be very different. For example, it is known that parental feeding styles have a remarkable influence on childhood obesity. Some studies drew the conclusion based on a questionnaire on feeding style, some used one categorical variable to summarize the general type, while some used calorie-intake or sugar-intake. Thus, if a region would like to design the customized policy or intervention that fits the local environment the best, it is difficult to make the decision which indicator can represent a general aspect most accurately, then it is unclear how to design corresponding interventions. In this case, applying machine learning models with local comprehensive data can help identify the most influencing variables grounded in real-world data, which can reduce the cost of testing factors and accelerate the establishment of policies or interventions that are more “evidence-based”. In addition to assisting on the localization of scientific research derived knowledge-based policy making, machine learning techniques can help confirming, with a wider real-world data, the results of scientific rationale and research for a specific region or timeframe, and occasionally derive insights or new hypothesis to be tested by scientific research and be candidate for adoption by policy decision making (if it’s validated and relevant). Therefore, it is expected that machine learning models can contribute to further scientific research as wells as to provide supports for policy decision making, providing domain experts with an additional tool to support their decisions making processes.

To reduce the features of the data, two main classes of machine learning models can be applied, namely feature extraction methods and feature selection methods. The difference between the two types of models is that feature selection methods keep a subset of the original variables while feature extraction methods combine the original variables into a smaller set of new features. In our study, we focus on feature selection methods to preserve the semantics of the features, as the results need to be interpretable without a subjective definition of new features. Moreover, it is easier for clinicians and policy-makers to establish follow-up interventions for a single feature than a compound factor of multiple features.

There are three main types of feature selection methods: filter methods, wrapper methods, and embedded methods [9]. Filter methods select features based on a statistical measure to assign a score to each variable to rank the variable importance regardless of the model. These methods are time-efficient and robust to overfitting, but tend to ignore the possible interactions between variables. Wrapper methods have the opposite advantages and disadvantages by converting the feature selection task into a search problem. Subsets of variables are compared with other subsets to select the group of features that can give the best predictive performance. Embedded methods are learning models that can perform feature selection and classification simultaneously by integrating feature selection algorithm as part of the learning process. The embedded methods take variable interactions into consideration and are less computationally demanding than wrapper methods. However, in some cases, the optimal feature set selected by one embedded method is classifier-dependent, meaning that the optimal set only works for this specific classifier and cannot contribute to good prediction when used for other embedded classifiers [10], as the optimal set is based on the hypotheses of the classifier.

Different feature selection methods have their specific advantages and disadvantages, resulting in the discrepancy in the selected variables. Hence, the results may be biased because of the model limitations if we only rely on one method. This problem is even more critical in exploratory research when the problem is not clear and the validity and credibility of the results are crucial. In addition, most feature selection methods, such as wrapper methods and embedded methods, select features based on predictive performance, which may lead to a selection of variables with no clinical relevance and interpretability. Finally, scalability of the model, i.e. the capability of handling a growing amount of data, is very essential when using a large-scale sample representative of the population. This means special attention is required in terms of time efficiency, data storage, data loading, and so on. Especially in our use case, it was not possible to load the whole dataset in memory at once, so it was an essential demand to develop a model with good scalability.

To overcome these limitations and solve the problems in a real-world setting, we propose BFSMR (the Bagging-based Feature Selection framework integrating MapReduce), a machine learning method that can perform efficiently with large-scale data and combine the results from different feature selection methods to give a more convincing and interpretable selection of features. A higher clinical relevance and a better interpretability of the algorithm-identified risk factors can give suggestions that are easy to implement in practice, which helps to better shape policies and corresponding interventions to overcome a specific public health challenge. Moreover, when using multiple feature selection methods as components of BFSMR, we can get a comprehensive understanding of the potential important risk factors preferred by different models, meaning that we are providing comprehensive information that is helpful for the decision-making process.

## Methods

### Participants and data source

This study uses data from the Osakidetza databases of the Public Health Provider in the Basque Country (Spain) [11], which provides services to more than 2,200,000 patients, through 16 hospitals and more than 300 primary health centers. This is a database recording information about patients for a global view of health status, not specifically for childhood obesity. The Osakidetza database was properly anonymized and extracted with the approval of an ethic committee in the Basque Country.

The database contains the information from around 400,000 children who were under 18 years old during 2000–2019 throughout the Basque Country and includes information of these children until they turned 18 years old. Three datasets from the database have been used for this study: children’s information table, children’s forms and the children’s mothers’ forms. Within Osakidetza, forms are health and lifestyle questionnaires that the GP or the nurse may or may not fill out in a consultation, recording the status of patients at that time point. Questions in these forms cover different aspects of lifestyles and health status, including physical exercise, smoking habits, drinking habits, diet styles, vegetable consumption, breastfeeding, gestational information, perinatal information, disease history and family disease history, etc.

**Fig. 1:**
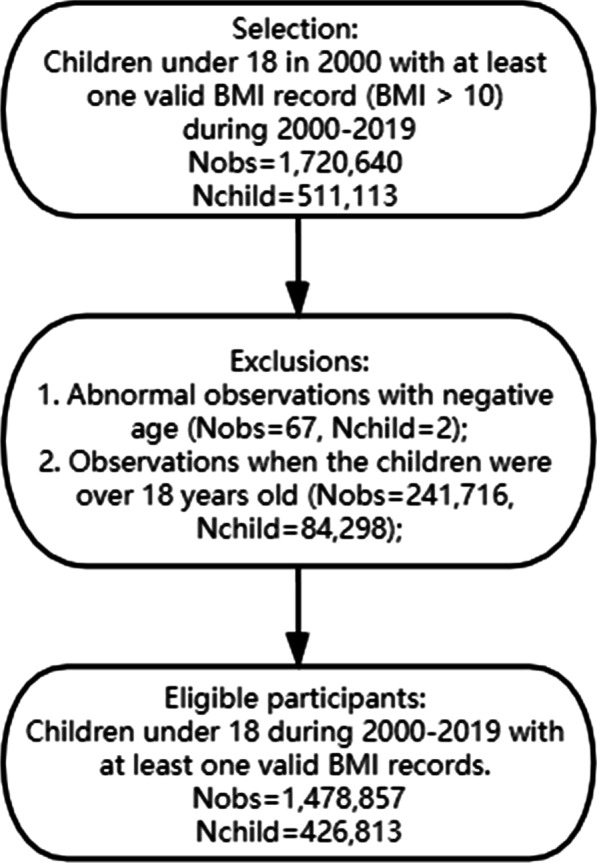
The selection and exclusion criteria for the participants

The original information was stored in 5 separate datasets, the cleansing and merging process is explained in detail in Additional file 1: Appendix A.1 and the final merged data has 1,478,857 records from 426,813 children (Selection and exclusion criteria are shown in Fig. 1). The multiple records from the same child disperse in an age span of 1–18 years old, representing different stages of childhood. Frequent repeated measures during a short period were rarely observed, therefore the personal-level longitudinality was not taken into consideration in the algorithm, instead, all the records were regarded as separate records. Besides, there were in total 119 variables used for feature selection, including numeric variables and dummy variables generated from categorical variables. Data pre-processing is detailed in Additional file 1: Appendix A.2. Due to the fact that in children’s forms different input variables are requested depending on the primary care visit motivation, missing rates varied based on the types of variables. The numeric variables about basic health status, such as blood pressure, age, mother’s BMI, child’s birth height and weight, etc., had very few missing values. While other numeric variables focusing on disease risks or life styles had much more missing values, as these questions were asked only when they were applicable. The missing rates for these variables varied between 30 and 70%. Most of the categorical variables also had a missing rate within this range, with very limited categorical variables having extremely high missing rate (over 80%), such as Diet Intent Change and Breastfeeding Abandonment.

### Outcome indicator

The indicator of childhood obesity was created based on the age- and gender-specific BMI. The European Childhood Obesity Group defines it as obesity if the BMI is higher than the 97th percentile of the age- and gender-specific subgroup in the reference population. A BMI higher than the 90th percentile is defined as overweight [12]. This study is aimed to observe both overweight and obese state and the 90th percentile is included in our statistics [13], so our outcome is to indicate whether the child has a BMI higher than the 90th percentile of the reference group.

### Embedded feature selection methods

The BFSMR is a bagging-based feature selection framework integrating MapReduce, which is a method with a balance between valid results and good interpretation. In this section, we first introduce the MapReduce technique, the bagging method, and the feature selection models used in the bagging framework. Then we construct a new framework incorporating the advantages of MapReduce and bagging at the same time.

#### MapReduce

MapReduce is a method to process and generate large-scale data in a parallel and distributed way, which can be very useful in the context of feature selection when the dataset is large-scale and high-dimensional. After splitting the input data into smaller subsets, the model extracts the information of interest in each subset and then merges them to output the aggregated results, ensuring to process large-scale data rapidly. To make it easier to understand, this can be summarized as “split-apply-combine” strategy.

The whole procedure can be broken down into two main tasks, Map and Reduce [14]. The original data is split into an appropriate size and each split is assigned with one Map function defined with respect to data structured in (key, value) pairs. The Map function works in parallel to convert every pair in the input data, denoted as (k1, v1), into a list of pairs in a different data domain, denoted as (k2, v2). Next, all the pairs (k2, v2) with the same key are collected to form one group for one key. Then the Reduce function is applied to each group in parallel and the collection of all Reduce calls is the final result. The procedures of splitting and mapping makes it possible to process the data in parallel and the procedures of shuffling and reducing merge the information by key variable to reduce the data scale.

$${\text{Map}}\left( {{\text{k}}1,{\text{v}}1} \right) \to {\text{list}}\left( {{\text{k}}2,{\text{v}}2} \right)$$

$${\text{Reduce}}\left( {{\text{k}}2,{\text{ list}}\left( {{\text{v}}2} \right)} \right) \to {\text{list}}\left( {\left( {{\text{k}}3,{\text{v}}3} \right)} \right).$$

#### 1.1.1. Bootstrap aggregating (bagging)

Bootstrap aggregating, also called bagging, is an ensemble-learning algorithm that applies different models with different random samples and uses majority voting to combine results for the final decision [15]. The method is incorporated in our model to merge results from different feature selection methods. Given a training data *D* of size *N* with correct labels $\omega_{l} \Omega = \left\{ {\omega_{1} , \ldots ,\omega_{C} } \right\}$ representing *C* classes, generate *T* bootstrapped samples *D*_*t*_ of size *n* by random sampling from *D* uniformly and with replacement. The model *m* can be applied with *D*_*t*_ to construct the classifier *h*_*t*_. With the ensemble $\varepsilon = \left\{ {h_{1} , \ldots ,h_{T} } \right\}$ derived from the training process, the unlabeled instance **x** in the testing data is classified into the class that receives the highest total vote.

#### Feature selection methods

In this study, we selected five models as representative methods from filter, wrapper and embedded methods to give a relatively comprehensive discussion and comparison of the feature selection methods. However, it is not necessary to always use the same feature selection methods. The applied methods can be determined based on the concrete problem and data issues.

We chose Mutual Information (MI) as the statistical measure for filter methods [16], and applied SVM-RFE as one type of wrapper methods [17]. In addition, we used 3 different models from embedded methods, namely Lasso Regression [18], Ridge Regression [19], and Random Forest [20]. Multiple embedded methods were used, because embedded methods have very different characteristics, making it difficult to use one to represent the diversity of embedded methods. We selected these methods because they are representative methods of filter, wrapper, and embedded method, and they are often discussed in other feature selection studies.

#### BFSMR

With all the models and techniques introduced above, we propose a framework that combines the advantage of MapReduce and Bagging and gives a more reasonable set of selected features with better interpretability. The notations used in this section are listed as below.

**Table:** 

Notation	Meaning
$D = \left\{ {d_{p} } \right\}$	*D* is the input data and is split into *P* chunks, denoted as $d_{p}$,$p = 1, \ldots ,P$
$c_{i}$	Feature selection classifier where $i = 1, \ldots ,M$
*cid*	The Classifier ID where $cid \in \left\{ {1, \ldots , M} \right\}$
*s*	Random sample set with Set ID $sid \in \left\{ {0, 1, \ldots , M} \right\}$ (*sid* = 0 for test set)
$w1_{j}$	Feature weights based on the ranking from each classifier where $j = 1, \ldots ,k$
$w2_{i}$	Method weights based on the model performance where $i = 1, \ldots ,M$
$f_{i}$	Feature lists derived from *M* feature selection classifiers,$f_{i} = \left\{ {f_{ij} ,j = 1, \ldots ,k} \right\}$
*F*	Feature space with unique features from *M* feature sts,$F = \left\{ {f_{l}^{^{\prime}} , l = 1, \ldots ,L} \right\}$
$V_{l}$	Voting score for each unique feature where $l = 1, \ldots ,L$

**Fig. 2:**
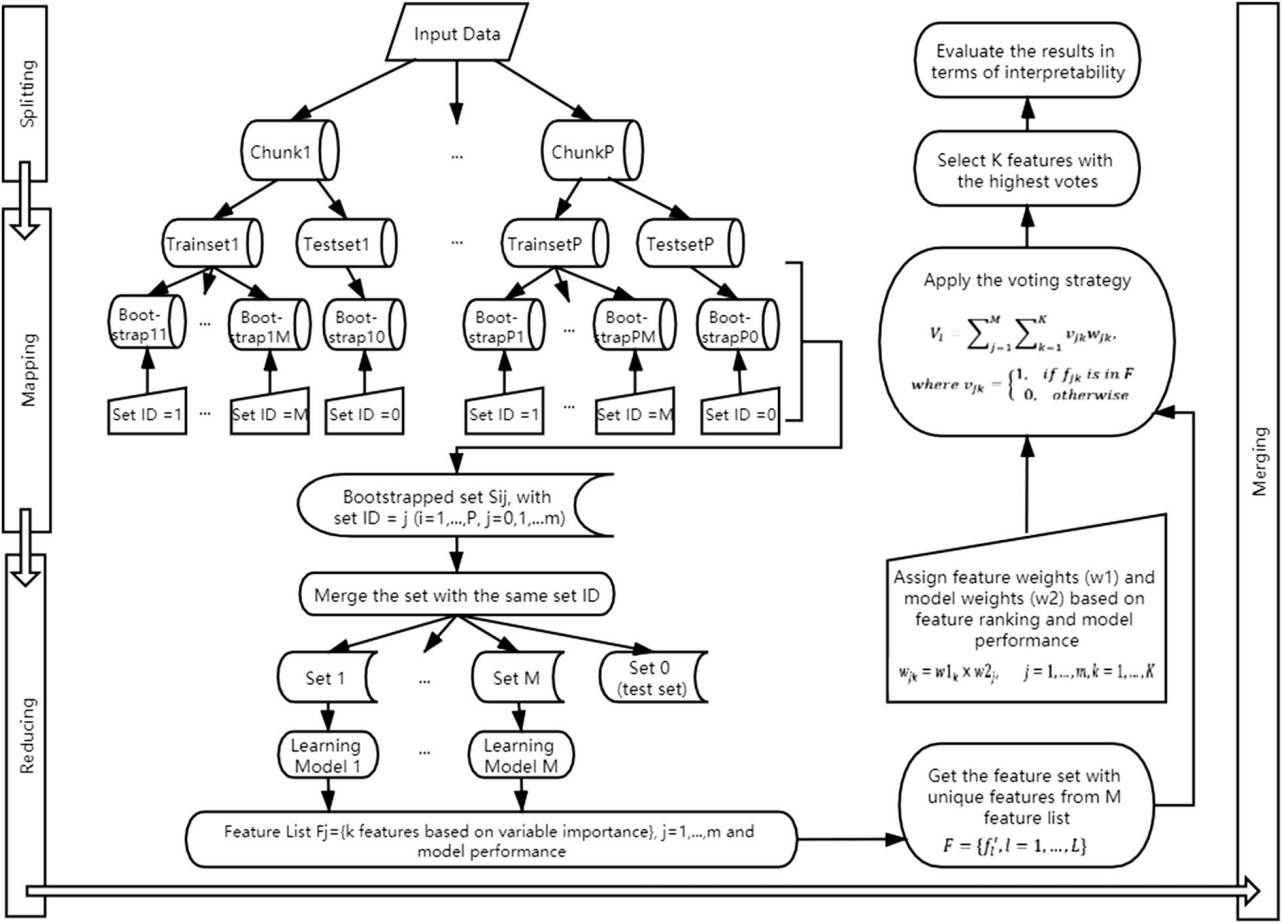
The main procedures of BFSMR. There are mainly four steps, (1) splitting, (2) mapping which draws bootstrapped samples from each chunk, (3) reducing which merges sets with the same Set ID and applies one feature selection classifier on each set and derive one selected feature list, and (4) merge procedure which combines the selected feature lists into the final output

The whole structure of BFSMR is shown in Fig. 2. After splitting the data into chunks, the Map function is merged with the bootstrapping procedure in Bagging. For each chunk, first it is randomly split into trainset and testset. Given *M* learning models, *M* bootstrapped samples are drawn from the trainset with a set ID *sid*. In addition, a testset is drawn (*sid* = *0*) without being influenced by any process in the trainset.

The sets with the same *sid* are merged to be used as the inputs of the Reduce function. The Reduce function works in parallel to each group. The original MapReduce normally applies the same model or function to all groups, but in BFSMR, we match the models with groups based on *sid* and model ID, so that the different model is applied to corresponding groups, which guarantees the possibility of using different feature selection methods to avoid model bias. The outputs of Reducing are *M* feature lists with *K* features selected by *M* models, and the model performance. A feature set *F* is the union of *M* feature lists.

Voting strategy is applied to merge the outputs from different models. The voting strategy of Bagging is majority voting with equal probability while we assign feature weights (*w1*) based on the ranking and assign model weights (*w2*) based on their predictive performance. The joint weight is calculated as below. Voting with weights is calculated (Equation as below) and the top *K* features with the highest votes are selected as the final results.

$$w_{jk} = w1_{k} \times w2_{j} , \quad j = 1, \ldots ,M, k = 1, \ldots ,K$$

$$v_{l} = \sum\limits_{j = 1}^{M} {\sum\limits_{k = 1}^{K} {v_{jk} w_{jk} } } ,\quad where \,\,\,\,v_{jk} = \left\{ {\begin{array}{*{20}l} {1,} \hfill & {{\text{if}}\,\,\, f_{jk} \,{\text{ is}}\,{\text{ in}}\, F} \hfill \\ {0,} \hfill & {{\text{ otherwise}} } \hfill \\ \end{array} } \right..$$

### Experimental setup

The data was imported in chunks with a size of 10,000 rows and we got 148 chunks in total with 147 full chunks and the last chunk only including 8857 rows. The split ratio of trainset and testset was 0.8:0.2 and the size of bootstrapped random samples was 10% of the trainset.

The five feature selection methods were applied in parallel and we selected 10 features from each method. We used the nearest-neighbor method to estimate MI in the Filter method (number of neighbors = 3) [16]. The features that had the maximum MI with the outcome, were regarded as features with the highest importance. To avoid the problem of long execution time, linear SVM was applied as the estimator for SVM-RFE and the absolute value of the coefficient was the feature importance. The step of the RFE method was set to 1, meaning that one variable was dropped in each iteration and the final 10 variables left in the model were the selected results. To determine the regularization parameter ($\lambda$) for Lasso and Ridge regression, the models were iteratively fitted along the regularization path on a grid of parameter, and the parameters that led to the best performance in the cross-validation test were selected, which was 0.002237 for the Lasso regression and 10 for the Ridge regression. As for the Random Forest, we used 50 estimators when training the classifier and used Gini impurity to measure the quality of a split [20]. The most commonly used feature importance is Mean Decrease in Impurity (MDI), however, impurity-based importance is biased towards numeric features or categorical features with high cardinality [21]. To overcome this limitation, we used permutation importance for feature evaluation [21]. All models were tested on the same testset. As the Filter method selected features without learning algorithms, linear regression was applied using the selected features to get the predictive performance.

The model applies the voting strategy that takes both feature weights and method weights into consideration. The feature ranking from the individual feature selection method was assigned as feature weights (*w1*). The five models were classified into 3 ranks based on model performance (details in Results) and the model weight was set as 1, 0.5 and 0.2 for each rank respectively (*w2*). To examine whether this voting strategy could effectively select the feature set with better interpretability and clinical relevance, it was compared with another two voting strategies and the selected results were compared [22–24]:

- **Voting1**: Voting with equal score for all features.
- **Voting2**: Voting with feature weights.
- **Voting3**: Voting with both feature and method weights.

### Evaluation measures

It is common to use performance measures to evaluate a learning system, however, in our use case, we would like to mainly evaluate the results in terms of interpretability.

There is no consensus about what interpretability is in machine learning at the current stage [25]. The results of feature selection methods are the potential risk factors of a disease, which should already be self-explanatory without an explanation method. However, it still makes difference if features have better clinical relevance and are easier to interpret and implement in practice.

To our knowledge, there is no discussion about the dimensions of interpretability of algorithm-identified features. The definitions, properties, and requirements of AI-explanations can show some potential dimensionalities. Properties of explanations which could lead to good interpretability include comprehensibility, certainty, importance, novelty, and representativeness [26], and human-friendly characteristics of explanations are contrastiveness, selectivity, social, inclination to abnormal, truthful, consistent with prior belief, general and probable [27].

After summarizing others’ work, we defined the dimensions of interpretability of algorithm-identified risk factors and evaluate the interpretability in Discussion.

## Results

Table1 presents the top 10 features selected from different models. Some variables had negative effects on the outcome indicators, meaning that higher values in the variables indicated lower risk of obesity. These variables are noted by (-) in Table1. There are some common features, such as age, sex, and mother’s diet education. However, the different model preference could still be observed. Lasso and Ridge were the specializations of linear regression with different regularization method, thus, they selected similar features, including smoking habits, exercise habits, and diet knowledge. Filter showed a preference for numeric variables (9 out of 10) and they were also variables with fewer missing values. Similarly, Random Forest had an inclination to numeric variables (4 out of 10) and variables with fewer missing values (Sex), but it also selected variables about smoking habits, exercise habits, and alcohol use. SVM-RFE selected the most different set from the others. Although it also covered diet information and Mothers’ exercise habit, the choices of the exact variables were different.

**Table 1:** Top 10 features selected from different models based on variable importance

	Filter (MI)	SVM-RFE	Ridge	Lasso	RandomForest
1	Age	MoDietEducation	Age	Age	SystolicPressure
2	Sleep_Normal (–)	MoRDType_LowSalt	Sex (–)	Sex (–)	MoDiastolicPressure (–)
3	BFType_Maternal (–)	RDType_2000 cal	Tobacco_No (–)	Tobacco_No (–)	MoSystolicPressure (–)
4	DiastolicPressure (–)	AdeDKnowledge	DietEducation	DietEducation	Sex
5	MoSystolicPressure	MoPE_Inadequate (–)	MoTobacco_Yes	MoDietEducation	Birthyear (–)
6	MoNumberCigarettes	DietCompliesAdvice	BFType_Maternal (–)	BFType_Maternal (–)	Tobacco_No (–)
7	Birthheight (–)	MoRDType_Free (–)	PE_Inadequate	Birthyear (–)	MoExerciseAdvice (–)
8	MoBMI	MoPEHour	MoDiabetes_No (–)	MoNumberCigarettes	MoAlcohol_No (–)
9	Birthweight (–)	DiastolicPressure (–)	PE_Adequate(–)	PE_Inadequate	PE_Inadequate
10	MoDiastolicPressure (–)	SystolicPressure	MoDietEducation	DCExecution _No	MoTobacco_Ex

All “Mothers-” in the variables were replaced with “Mo-” for shorter names

RDType, RecommendedDietType; MoRDType, MoRecommendedDitetType; BFType, BreastfeedingType; PE, PhysicalExercise; MoPE, MoPhysicalExercise; MoPEHour, MoPhysicalExerciseHour; AdeDKnowledge, AdequateDietaryKnowledge; DCExecution, DietCorrectExecution,

The model predictive performance was evaluated on the same testset (Table2) based on accuracy and F-score. Ridge, Lasso and Filter had good performance with both measures, SVM-RFE performed at a moderate level as the F-score was not high, and Random Forest had the lowest scores of both measures. Although the performance of Filter was relatively good, it failed to consider variable interactions. Based on the performance and model property, the five models were classified into three ranks with different weights: (1) Rank1, Lasso and Ridge (weight = 1); (2) Rank2, Filter and SVM-RFE (weight = 0.5); (3) Rank3, Random Forest (weight = 0.2).

**Table 2:** Comparison of predictive performance among different models

	Filter (MI)	SVM-RFE	Ridge	Lasso	RandomForest
Accuracy	0.843	0.845	0.844	0.839	0.828
F Score	0.915	0.774	0.915	0.912	0.770

Accuracy and F-score were jointly used to evaluate the performance. Lasso, Ridge, and filter method had relatively better performance and Random Forest had the worst performance

**Fig. 3:**
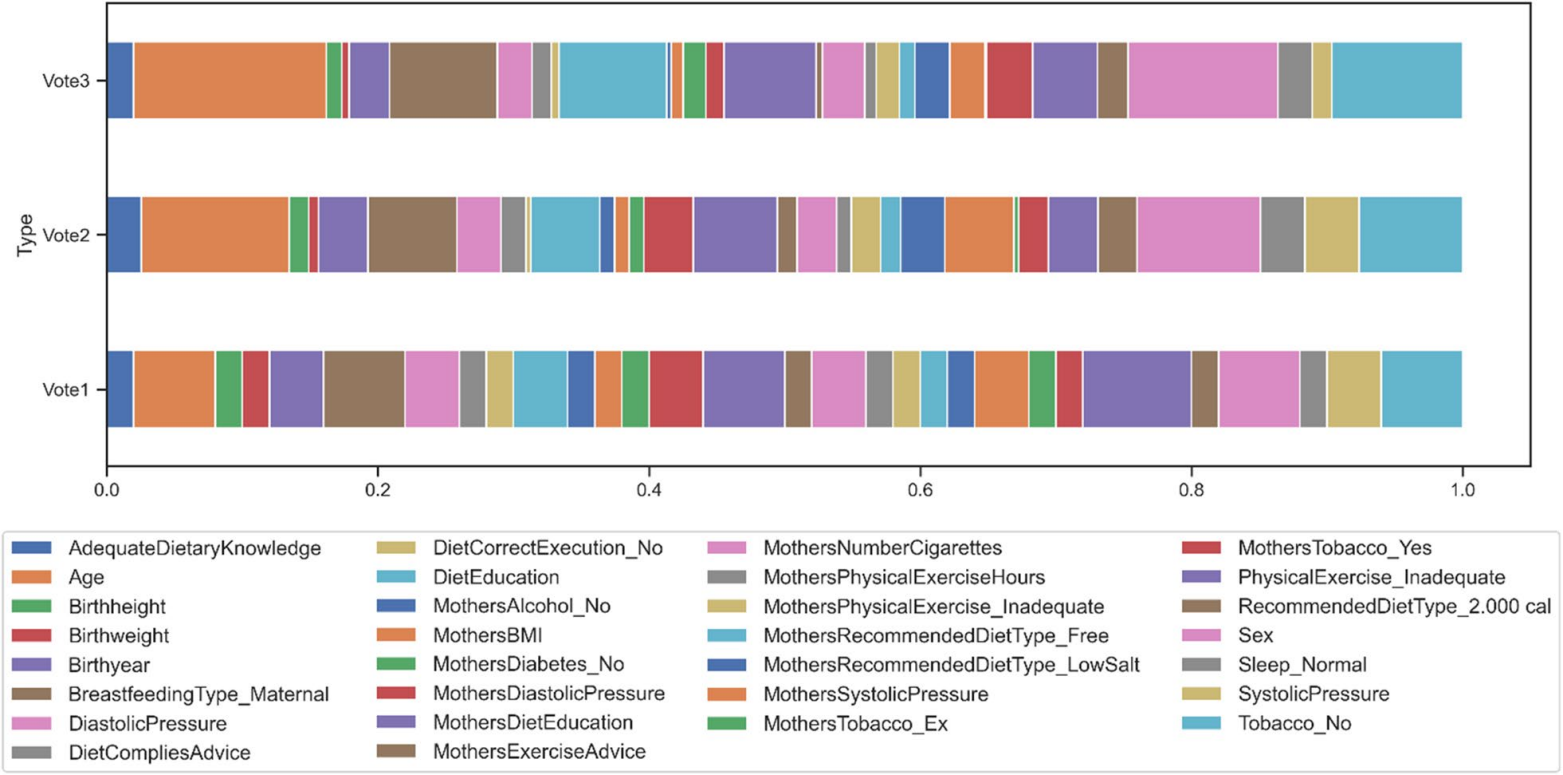
Visualization the comparison of selected variables with high scores calculated from 3 voting strategies. The plot was drawn using the percentage of the score of one feature out of the whole set and larger percentage means higher variable importance. Age, Sex, Tobacco_No, DietEducation, and BreaskfeedingType_Maternal gradually gained more importance during the changes from Voting1 to Voting3 while the importance of MothersDiastolicPressure and PhysicalExercise_Inadequate dropped. MothersDietEducation was more stable and took almost the same share of voting scores in all strategies

The voting scores for 3 voting strategies are shown in Table 3 and visualized in Fig. 3, which shows the percentage of the score of one feature out of the whole set. The voting scores can be regarded as the variable importance of this ensemble feature selection framework. Some features gradually gained more importance from Voting1 to Voting3, including age, sex, no smoking, child’s diet education, and maternal breastfeeding, which were the top 5 features selected by Voting3. In contrast, mother’s diastolic pressure lost its superiority. Inadequate physical exercise had a similar trend although it was still among the top 10 features of Voting3. Mother’s diet education was more stable and took almost the same share of voting scores in all strategies.

**Table 3:** Comparison of the selected variables with high scores calculated from different voting strategies

Voting1		Voting2		Voting3	
PE_Inadequate	4	Age	30	Age	25
Age	3	Sex	25	Sex	19.4
BFType_Maternal	3	Tobacco_No	21	Tobacco_No	17
MoDietEducation	3	BFType_Maternal	18	BFType_Maternal	14
Sex	3	MoDietEducation	17	DietEducation	14
Tobacco_No	3	DietEducation	14	MoDietEducation	12
Birthyear	2	MoSystolicPressure	14	PE_Inadequate	8.4
DiastolicPressure	2	SystolicPressure	11	MoTobacco_Yes	6
DietEducation	2	Birthyear	10	MoNumberCigarett	5.5
MoDiastolicPressure	2	MoDiastolicPressure	10	Birthyear	5.2
MoNumberCigarettes	2	PE_Inadequate	10	MoSystolPressure	4.6
MoSystolicPressure	2	DiastolicPressure	9	DiastolicPressure	4.5
SystolicPressure	2	MoRDType_LowSalt	9	MoRDType_LowSalt	4.5
AdeDKnowledge	1	Sleep_Normal	9	Sleep_Normal	4.5
Birthheight	1	MoNumberCigarettes	8	RDType_2.000cal	4
Birthweight	1	RDType_2.000cal	8	AdeDKnowledge	3.5
DietCompliesAdvice	1	AdeDKnowledge	7	MoDiabetes_No	3
DCExecution_No	1	MoPE_Inadequate	6	MoPE_Inadequate	3
MoAlcohol_No	1	MoTobacco_Yes	6	DietComplieAdvice	2.5
MoBMI	1	DietCompliesAdvice	5	SystolicPressure	2.5
MoDiabetes_No	1	Birthheight	4	MoDiastoPressure	2.3
MoExerciseAdvice	1	MoExerciseAdvice	4	Birthheight	2
MoPE_Inadequate	1	MoRDType_Free	4	MoRDTyp_Free	2
MoPEHours	1	MoAlcohol_No	3	MoBMI	1.5
MoRDType_LowSalt	1	MoBMI	3	MoPEHours	1.5
MoRDType_Free	1	MoDiabetes_No	3	Birthweight	1
MoTobacco_Yes	1	MoPEHours	3	DCExecution_No	1
MoTobacco_Ex	1	Birthweight	2	MoExerciseAdvic	0.8
RDType_2.000cal	1	DCExecution_No	1	MoAlcohol_No	0.6
Sleep_Normal	1	MoTobacco_Ex	1	MoTobacco_Ex	0.2

The top variables changed and the importance of some variables gradually grew with the change from Voting1 to Voting3

All “Mothers-” in the variables were replaced with “Mo-” for shorter names

RDType, RecommendedDietType; MoRDType, MoRecommendedDitetType; BFType, BreastfeedingType; PE, PhysicalExercise; MoPE, MoPhysicalExercise; MoPEHour, MoPhysicalExerciseHour; AdeDKnowledge, AdequateDietaryKnowledge; DCExecution, DietCorrectExecution

The top 10 features selected by BFSMR are age, sex, birth year, breastfeeding type, smoking habit and diet-related knowledge of both children and mothers, exercise, and Mother’s systolic blood pressure. The results indicate that smoking habit, lack of exercise, and unbalanced diet of both mothers and children are the risk factors of childhood obesity. Besides, boys have higher risk than girls and the risk grows along with age. It is also found that maternal breastfeeding can reduce the risk and younger generation (based on birth year) tend to suffer more from obesity.

## Discussion

### Model interpretability and clinical relevance

#### Clinical relevance and importance

The voting strategy played an important role in selecting a more reasonable feature set with better clinical relevance. When applying individual feature selection methods, there could be bias in the results caused by model limitation. For example, as shown in Table 1, Filter and Random Forest had an inclination to numeric variables and variables with fewer missing values. In Experimental setup, we explained that we used permutation importance for feature evaluation when applying Random Forest, as it was known that the commonly used feature importance, MDI, would be biased towards numeric features or categorical features with high cardinality. However, the results turned out that there was still bias in the results even if we used permutation importance. These results proved that there would be risks that categorical variables with high missing rates were disregarded if some feature selection models were applied individually. Our method, BFSMR, provided combined results from a set of feature selection methods, which could reduce or eliminate the bias affected by missing values.

Bagging uses the majority voting with equal probability (Voting1). But the close scores of Voting1 made it difficult to distinguish the most important ones. For example, 13 features had scores higher than 2, making it impossible to select only the top 10 features as the final output. Voting2 added feature weights based on the ranking from each model, nevertheless, variables with high rankings from poorly-fitted models could still affect the results. One significant difference between Voting2 and Voting3 was the notable decline in the rankings of the numeric variables of less relevance, e.g. mothers’ systolic pressure, the child’s systolic pressure. The relevant numeric variables were not negatively affected, on the contrary, the average number of mother’s cigarette consumption climbed from the 15th in Voting2 to the 9th in Voting3.

#### Easiness to implement in practice

BFSMR could select variables that were easier to implement in practice for the follow-up interventions or policies. For example, although three models (SVM-RFE, Ridge, Lasso) covered diet-related information, different variables were selected, e.g. diets with less than 2000 cal, whether follow the diet advice, whether correctly execute diet advice. These variables were concrete about one specific aspect but it would be difficult to use them to make corresponding interventions. The features selected by BFSMR were child’s diet education and mother’s diet education, which could be easily put in practice in real life, for example by arranging lectures at school or suggesting pregnant women taking specifically developed courses.

#### General and probable

BFSMR is a general framework to deal with large-scale data and combine results from multiple models. It is flexible because the individual feature selection methods applied in this framework are not strictly defined but can be replaced with other methods, and more methods of interest can be added into the framework. Therefore, it is probable to apply BFSMR in most use cases.

#### Representativeness

In the study, 5 models were chosen as representatives of filter, wrapper, and embedded methods, meaning that a wide range of model types were covered. Apart from the methods, the algorithm-identified risk factors are also representative risk factors that can well summarize one aspect, for example, child’s diet education is a good representative for diet-related suggestions.

#### Comprehensibility

Although the results of individual model were not reported as the final output, the process of applying multiple models as components of ensemble feature selection was a comprehensive exploration of important features. Some variables only appeared once in one model, which might be neglected if only one model was applied. For instance, Sleep_Normal had a negative effect in the Filter method, which was in agreement with clinical knowledge [28, 29]; mother’s BMI and mother’s diabetes disease history were reasonable and could indicate genetic influence; mother’s diet type (Low Salt) and mother’s diet type (Free) suggested the ideal diet styles. These variables were not included in the current output, but it would be very likely these variables were selected if we chose a higher number of selected features, for instance, Sleep_Normal and mother’s diet type (Low Salt) ranked 12th and mother’s diabetes disease history was at the 17th.

#### Consistency with prior beliefs

There are numerous studies on the risk factors for childhood obesity, which can be classified into two main types, the genetic factors and environmental factors [2]. This paper focused more on environmental factors, as it would be easier to make corresponding policies for environmental factors. This type of factors include lifestyle factors such as eating behaviors [30], sleeping pattern [28, 29], parental feeding styles [30]. The environmental factors may also include some other factors such as environmental chemicals or microbiota, but the database used in this paper does not include such information. A study in 2001 suggest that the main risk factors for obesity in children include dietary intake, physical activity, family characteristics, parents’ lifestyles and environmental factors such as school policies and demographics [31]. Another study in 2013 identified parent BMI, child sleep duration and parental restrictive feeding as the main risk factors [32]. A more detailed literature review of previous studies on risk factors of childhood obesity can be found in Additional file 1: Appendix B.1.

Based on the previous studies about the risk factors of childhood obesity (Additional file 1: Appendix B.1), the main causes include lifestyle factors such as eating behaviors, physical activity, sleep, age, gender, parents’ lifestyles, and smoking habit, which were all covered in our results. Some studies pointed out the relation with genetic factors and psychological factors, but such information was lacking in our data. In general, our results are consistent with prior beliefs.

### Contributions and limitations

One contribution of the paper is the distributed implementation for ensemble feature selection. Although some studies applied distributed implementation for individual methods [33], few papers tried it for ensemble feature selection. A homogeneous-distributed ensemble was proposed [34–36], which split the trainset into subsets and applied the same method. However, using the same method on the divided subsets cannot avoid model bias. Thus, it is necessary to apply distributed implementation for ensemble feature selection when multiple feature selections are included. Moreover, MapReduce avoided full load, so that the real-world problem of platform limitation was solved.

Another contribution is the voting strategy. In general, there are three ways of combining the outputs of ensemble feature selection: combination of label predictions, features subsets, and features rankings [37]. The combination of label predictions are most widely used and the models are trained to achieve the best performance. However, this method cannot work well when the goal is to select a subset of features with clinical relevance. To solve the problem, some studies combine the results by having the intersection or union of the features from different selectors [38], and some studies incorporate feature rankings (Voting2) [39, 40]. Our voting strategy (Voting3) has both feature rankings and model performance, making it possible to select a more reasonable set. A detailed literature review is in Additional file 1: Appendix B.2.

Furthermore, no discussion was identified on the interpretability of algorithm-identified risk factors. To better evaluate interpretability, the dimensions of interpretability were defined and summarized in this paper.

Finally, previous studies showed that there were regional differences in childhood obesity trends [41], but there were no studies in more details on a regional level. Basque government’s goal within this study was to find out the risk factors of their own region and make corresponding policies.

One main characteristic of BFSMR is its flexibility. This paper used 5 feature selection methods, namely filter methods based on MI, SVM-RFE, Lasso Regression, Ridge Regression, and Random Forest. However, it is not necessary to apply these methods in all use cases and the number of models can be more or less than 5. The type of feature selection methods can be adjusted according to the specific research goal of the study. Similarly, the model weights can also be adjusted based on the model performance. This paper classified the models into 3 ranks, because it could be observed that the models were on high, moderate, and low levels in terms of performance evaluation. In a different use case, it could happen to evaluate 10 models, and to adopt a classification of 4 or 5 ranks or 4 models into 2 ranks (the technological approach is flexible). In fact, this ensemble feature selection method is similar to the process of formulating standards and industry guidelines by an expert committee. Each individual method acts as one expert and the model weight can be regarded as the priority of one expert’s opinion because of the expert’s past experience. Therefore, the model weight needs to be discussed case by case based on the real situation, so that we don’t give a strict requirement of the number of ranks or the value of weights. The core values of BFSMR is the distributed implementation for ensemble feature selection method and the voting strategy to merge the final results.

Most clinical studies of childhood obesity focused on smaller age groups to give precise conclusions [42, 43]. Our participants had a large age span, aiming for better policy making. We defined the outcome indicator on a population level, targeting on policy for all children, instead of a specific age group. It is acceptable, as the policy is normally to adjust general behavioral patterns that can have long-term effects, and our results could well serve this purpose. The current results are for Basque Country, subgroup analyses will be done for province-level and town-level data and it will eventually go to small villages.

One limitation of this study is the predictive performance of the five models was not very satisfactory due to the sparse structure, missing values, and the small number of selected features. The model performance can be further improved if more features are kept, or by using methods that are specifically for sparse data. Another limitation is causality. The current study, same as other classical feature selection methods [44], selected features based on correlation, and the models were diagnostic models instead of prognostic prediction models [45]. However, it could be solved if the individual method within the framework was causality-based models or prognostic models. In summary, BFSMR acts as a general strategy to provide the framework of a meta-algorithm. The feature selection models can be replaced if particular data issues need to be solved. In terms of generalization, one limitation of this study is that all the participants were the children from Basque region. Previous studies showed that there were regional differences in childhood obesity trends [41], thus, the generalization of the identified risk factors to other populations should be cautious.

## Conclusion

We presented a new ensemble feature selection framework that combines MapReduce and Bagging to make it possible to deal with large-scale data and applies 5 feature selection models to avoid model bias. A collection of risk factors of childhood obesity with better interpretability and clinical relevance were identified, which solved the practical research question raised by the department of health, to contrast with their experience and knowledge and provide supports for the decision-making of future interventions and policies. The proposed framework can also be applied to select risk factors of other chronic conditions in the future.

## Supplementary Information

**Additional file 1.** Data Processing and Related Work.

## Data Availability

The data that support the findings of this study are available from BIOEF but restrictions apply to the availability of these data, which were used under license for the current study, and so are not publicly available. Data are however available from the authors upon reasonable request and with permission of BIOEF.

## Abbreviations

BFSMR: The Bagging-based feature selection framework integrating MapReduce
MI: Mutual information
