# Supplementary material for: An ensemble-based feature selection framework to select risk factors of childhood obesity for policy decision making
Source: BMC Med Inform Decis Mak. 2021 Jul 21;21:222. doi: 10.1186/s12911-021-01580-0 (PMC8293582; doi:10.1186/s12911-021-01580-0)
Supplement: Supplementary file 1 — Additional file 1. Data Processing and Related Work. [file 12911_2021_1580_MOESM1_ESM.docx]

APPENDIX A. Data pre-processing

A.1 Data cleansing and harmonization

The Osakidetza dataset is composed of data extracted from primary care, specialty care and hospital databases, including information about the patient, diagnosis, medical forms, medical appointments, prescription information, and information about the child’s mother. There are 9 datasets in Osakidetza. The datasets extracted from Osakidetza needed cleansing since they had inconsistencies, empty columns, and incorrect formatting values. In addition, data about the forms were originally stored in five different datasets: three about children’s forms taken from primary care, specialty care and nurse collection records respectively and two about mothers’ forms taken from primary care and specialty care.

First, data harmonization was conducted as different form sources contain variables that refer to the same concept but expressed in different ways and/or in different units (e.g. the “weight” variable was expressed in grams on primary care forms and the “weight” variable was expressed in kilograms on specialty care forms). Second, the five forms datasets were merged into two datasets (i.e. children’s forms and mothers’ forms) based on childrenID and motherID. Third, the mother’s forms data was aggregated by year by using the mean values for the numeric variables and the mode values for the categorical variables and dummy variables with values of 0 and 1. The variables of sex, birthdate, and the child’s mother’s ID in children’s information table were added into children’s form data and this merged table was further joined with mother’s aggregated forms data based on motherID and registration year in both tables. In this case, the mother’s forms data becomes additional variables for the children to provide maternal and hereditary information. Finally, the empty columns were dropped and rows with empty BMI were not useful for our study. In the end, only records of the children under the age of 18 with children’s BMI in the range between 10 and 60 were kept.

The final merged data has 1,478,857 records from 426,813 children.

A.2 Data pre-processing

The outcome was defined based on BMI. In fact, other variables such as waist are also used as indicators in obesity-related studies,[1] which will show strong correlation with our outcome if these variables are included in the predictors. In this case, 5 variables were dropped, i.e. BMI, height, weight, waist, and size.

There are three types of variables per record: 28 numeric variables, 8 dummy variables, and 33 categorical variables. Almost all variables have missing values. We replaced missing values with 0 for the numeric variables and with “Unclear” for categorical variables. With the refilled “Unclear”, the *n*-level categorical variables were converted into *n+1* dummy variables by using One-Hot encoding.[2] To overcome the limitation of One-Hot encoding, the dummy of “Unclear” was dropped to avoid the multicollinearity.[3] It was then used as the default option for each variable. Eventually, 83 dummy variables replaced 33 categorical variables, with each dummy representing one level of the categorical variables, denoted as “VarName_LevelName”. The exception was the “Yes” dummy of the two-level categorical variables, denoted as “VarName”. Both “Yes” and “No” dummy were kept for the two-level categorical variables, because there were a considerable number of “Unclear” observations, meaning that “Yes” and “No” didn’t represent the same information and it would not cause multimorbidity when keeping both of them. It was tested to process the eight dummy variables in the same way as categorical variables. However, it would lead to severe multicollinearity in the data because the dummy variables had limited negative observations. In this case, we only kept the dummy representing the positive answers. All the data was standardized before model application. Finally, we had 119 variables in total and the detailed variable names can be found in Table A.1.

**Table A.1.** A full list of features used in the experiment.

| Numeric Variables (28) | Birthyear,Age,SystolicPressure,DiastolicPressure,Glucose,Triglycerides,UricAcid,Gpt,Got,FruitsPerDay,FruitVegetableConsumption,NumberCigarettes,AlcoholUnitWeek,Birthheight,Birthweight,GestationalAge,CardiovascularRisk,FraminghamCardiovascularRisk,RegicoCardiovascularRisk,MoSystolicPressure,MoDiastolicPressure,MoBMI,MoAlcoholUnitWeek,MoNumberCigarette,MoCardiovascularRisk,MoFraminghamCardiovascularRisk,MoRegicoCardiovascularRisk,MoPhysicalExerciseHours |
| --- | --- |
| Dummy Variables (8) | Sex,DietIntentChange,DietCompliesAdvice,MoRecommendedDiet,MoBirth,MoGestationalDiabetes,MoDietIntentChange,MoUnknownVariable |
| Categorical Variables (33/83 ) | **YES/NO**(19/38):Alcohol,Diabetes,ExerciseAdvice,BreakfastDairy,BreakfastFruit,DietCorrectExecution,AdequateDietaryKnowledge,MoAlcohol,MoExerciseAdvice,MoFitnessAdvice,MoDiabetes,MoDietCorrectExecution,MoAdequateDietaryKnowledge,MoBirthPreparation,MoBreastfeedingEducation,MoMaternalBreastfeedingInformation,BreastfeedingAbandonment,DietEducation,MoDietEducation;  **Normal/Abnormal**(1/2):Sleep;  **Adequate/Inadequate**(4/8):Diet,MoDiet,PhysicalExercise,MoPhysicalExercise;  **Multiple categories** (9/45):Tobacco(6),MoTobacco(6),MoSmoker(2),RecommendedDietType(6), MoRecommendedDietType(8),TypeBreastfeeding(4),MoTypeBirth(6),MoPlaceBirth(4),MoPromotionBreastfeeding(3) |

Note: All “Mothers-” in the variables were replaced with “Mo-” for shorter names.

Reference

1. Jacobsen B, Aars N. Changes in waist circumference and the prevalence of abdominal obesity during 1994–2008 - cross-sectional and longitudinal results from two surveys: the Tromsø Study. *BMC Obes* 2016;3:41.
2. Harris D, Harris S. Digital design and computer architecture. San Francisco, CA: Morgan Kaufmann 2012:129.
3. Garavaglia S, Sharma A. A Smart Guide to Dummy Variables: Four Applications and a Macro. In: Proceedings of the Northeast SAS Users Group Conference 1998.

APPENDIX B Related Work

B.1 Risk factors of childhood obesity

There are numerous studies on the risk factors for childhood obesity. In general, obesity is often considered to be the result of an imbalance between calories taken in and burned out. However, there is increasing evidence that other factors such as genetic background play a key role in determining the risk of obesity.[1] According to the review in 2017,[2] childhood obesity is the result of an interaction between different factors such as the environment, genetics and a child’s surroundings. Environmental factors include lifestyle factors such as eating behaviors, which is highly related to parents’ feeding styles, physical activity, stress and depression.[3] Other major environmental factors include perinatal factors,[4-5] birth size,[6] catch-up growth,[7] environmental chemicals,[8] microbiota,[9-10] and adverse life experiences.[11] There are some studies that have demonstrated an association between the sleep time duration and obesity.[12-13]

A study in 2001 suggest that the main risk factors for obesity in children include dietary intake, physical activity and sedentary behaviour, moderated by factors such age and gender. In addition, family characteristics, parents’ lifestyles and environmental factors (e.g. school policies and demographics) have a major impact in children’s lifestyle and, therefore, their risk of obesity.[14] Another study in 2013 used multiple regression analyses to identify childhood obesity risk factors from data collected in a longitudinal study of preschool children and they conclude that the three early-life risk factors are parent BMI, child sleep duration and parental restrictive feeding.[15] Hammond’s research predicted childhood obesity using electronic health records and publicly available data by means of a variety of machine learning algorithms.[16]

It is expected that the selected features of our framework could be in corresponding to previous studies, proving the reliability of the results and the possibility of using the framework to select risk factors in the future study.

B.2 Combination of the results for ensemble feature selection

In general, there are three ways of combining the outputs of ensemble feature selection: combination of label predictions, combination of subsets of features, and combination of rankings of features.[17] The combination of label predictions are most widely used in studies, which use techniques such as majority voting to select the most likely class labels from the class labels predicted by all classifiers, or use decision rules to decide the final class label if the classifiers output a degree of certainty.[18] The final outputs are the predicted class labels, and performance measures, e.g. accuracy or precision, are often used to evaluate the results.

However, in some cases, it is expected to have selected features as the output and the goal is to select a subset of features that are diverse, stable, and interpretable. If classification is still necessary, it can be applied as an additional procedure. The most typical way of combining the subsets of features is to use the intersection or union of the features selected by different selectors.[19] Some studies incorporated decision rules when making unions, e.g., they only included the new subsets into the union when the new subsets could improve the classification accuracy,[20] or data complexity.[21]

The last way of combining the results is to combine the rankings of the features, as some feature selection methods are capable to output an ordered feature list, instead of a set of features. The rankings of the features can be aggregated by using some simple statistics, such as minimum or median. Some studies improved the aggregation method by using order statistics.[22-23] They compared the actual rankings with the expected behavior of uncorrelated rankings, and then re-ranked the features and calculated significance scores. However, this method requires simulations to define significance thresholds and does not support partial rankings, meaning that the rankings must contain all the features.

The last two methods of combining results share similarity as both of them return the selected features instead of the predicted labels. Therefore, it is worth to try a joint method to take the advantages of these two methods.

Reference

1. Sahoo K, Sahoo B, Choudhury A, et al. Childhood obesity: causes and consequences. *J Family Med Prim Care* 2015;4(2):187-92.
2. Kumar S, Kelly A. Review of Childhood Obesity. *Mayo Clinic Proceedings* 2017;92(2):251 – 265.
3. El-Behadli A, Sharp C, Hughes S, et al. Maternal depression, stress and feeding styles: Towards a framework for theory and research in child obesity. *British Journal of Nutrition* 2015;113(S1):S55-S71.
4. Davis E, Lazdam M, Lewandowski A, et al. Cardiovascular Risk Factors in Children and Young Adults Born to Preeclamptic Pregnancies: A Systematic Review. *Pediatrics* 2012;129(6):e1552-e1561.
5. Lau E, Liu J, Archer E, et al. Maternal weight gain in pregnancy and risk of obesity among offspring: a systematic review. *J Obes.* 2014;2014:524939.
6. Yu Z, Han S, Zhu G, et al. Birth weight and subsequent risk of obesity: a systematic review and meta‐analysis. *Obesity Reviews* 2011;12:525-542.
7. Taveras E, Rifas-Shiman S, Sherry B, et al. Crossing Growth Percentiles in Infancy and Risk of Obesity in Childhood. *Arch Pediatr Adolesc Med* 2011;165(11):993–8.
8. Warner M, Wesselink A, Harley K, et al. Prenatal exposure to dichlorodiphenyltrichloroethane and obesity at 9 years of age in the CHAMACOS study cohort. *Am J Epidemiol* 2014;179(11):1312-22.
9. Kalliomäki M, Collado M, Salminen S, et al. Early differences in fecal microbiota composition in children may predict overweight. *The American Journal of Clinical Nutrition* 2008;87(3):534-538.
10. Chang L, Neu J. Early Factors Leading to Later Obesity: Interactions of the Microbiome, Epigenome, and Nutrition. *Current Problems in Pediatric and Adolescent Health Care* 2015;45(5):134-42.
11. Fuemmeler B, Dedert E, McClernon F, et al. Adverse childhood events are associated with obesity and disordered eating: results from a U.S. population-based survey of young adults*. J Trauma Stress* 2009;22(4):329-33.
12. Jiang F, Zhu S, Yan C, et al. Sleep and Obesity in Preschool Children. *The Journal of pediatrics* 2009;154(6):814-8.
13. Sekine M, Yamagami T, Handa K, et al. A dose–response relationship between short sleeping hours and childhood obesity: results of the Toyama Birth Cohort Study. *Child: Care, Health and Development* 2002;28:163-170.
14. Davison K, Birch L. Childhood overweight: a contextual model and recommendations for future research. *Obes Rev.* 2001;2(3):159-71.
15. Dev D, McBride B, Fiese B, et al. Behalf Of The Strong Kids Research Team. Risk factors for overweight/obesity in preschool children: an ecological approach. *Child Obes.* 2013;9(5):399-408.
16. Hammond R, Athanasiadou R, Curado S, et al. Predicting childhood obesity using electronic health records and publicly available data. PLoS One 2019;14(4): e0215571.
17. Bolon-Canedo V, Alonso-Betanzos A. Ensembles for feature selection: A review and future trends. *Information Fusion* 2019;52:1-12.
18. Peteiro-Barral D, Guijarro-Berdiñas B. A survey of methods for distributed machine learning. *Progress in Artificial Intelligence* 2013;2(1):1–11.
19. Alvarez-Estevez D, Sanchez-Marono N, Alonso-Betanzos A., et al. Reducing dimensionality in a database of sleep EEG arousals. *Expert Systems with Applications* 2011;38(6):7746–7754.
20. Bolon-Canedo V, Sánchez-Marono N, Alonso-Betanzos A. Distributed feature selection: an application to microarray data classification. *Applied Soft Computing* 2015;30:136–150.
21. Moran-Fernandez L, Bolon-Canedo V, Alonso-Betanzos A. Centralized vs. distributed feature selection methods based on data complexity measures. *Knowledge-Based System* 2017;117:27–45.
22. Stuart JM, Segal E, Koller D, Kim SK. A gene-coexpression network for global discovery of conserved genetic modules. *Science* 2003;302(5643):249–255.
23. Aerts S, Lambrechts D, Maity S, et al. Gene prioritization through genomic data fusion. *Nature Biotechnology* 2006;24(5):537.
